# Histopathologic tumor spreading in primary ovarian cancer with modified posterior exenteration

**DOI:** 10.1186/s12957-015-0647-x

**Published:** 2015-07-31

**Authors:** Kazuyoshi Kato, Kyoko Nishikimi, Shinichi Tate, Takako Kiyokawa, Makio Shozu

**Affiliations:** Department of Gynecology, Chiba University School of Medicine, 1-8-1 Inohana, Chuo-ku, Chiba 260-8670 Japan; Department of Molecular Pathology, Chiba University School of Medicine, 1-8-1 Inohana, Chuo-ku, Chiba 260-8670 Japan; Present address: Department of Gynecology, Cancer Institute Hospital, 3-8-31 Ariake, Koutou-ku, Tokyo 135-8550 Japan

**Keywords:** Ovarian cancer, Modified posterior exenteration, Histopathology, Primary debulking surgery, Interval debulking surgery

## Abstract

**Background:**

To achieve optimal cytoreduction for advanced-stage ovarian cancer, modified posterior exenteration is the most frequently performed bowel surgery. We assessed the extents of tumor spreading in the rectosigmoid wall and pelvic side wall in modified posterior exenteration specimens during primary debulking surgery (PDS) and interval debulking surgery (IDS) following neoadjuvant chemotherapy, and compared the validity of selecting this surgical procedure in the patients undergoing PDS with that in the patients undergoing IDS.

**Methods:**

Clinicopathological data from consecutive patients who had undergone a modified posterior exenteration for primary ovarian, tubal, and peritoneal cancer at our institution between April 2008 and March 2013 was retrospectively reviewed.

**Results:**

A total of 75 patients (38 in PDS and 37 in IDS) were included in this study. Tumor involvement of the rectosigmoid was histopathologically confirmed in 65 % of the specimens. Though the extent of tumor spreading in the rectosigmoid was deeper in PDS than in IDS, the frequency of tumor involvement of the rectosigmoid in patients who had undergone modified posterior exenteration during PDS was equivalent to that in the IDS group. Lateral tumor spreading to the side wall(s) was histopathologically confirmed in 53 % of the patients in whom a pelvic side wall resection had been performed.

**Conclusions:**

During both PDS and IDS for ovarian cancer presenting with tumor involvement of the cul-de-sac, close inspection and palpation by gynecologic oncologists may enable the extent of tumor spreading in the pelvis to be estimated, enabling valid decisions as to whether an en bloc resection of the pelvic tumors together with the rectosigmoid and the pelvic side wall might or might not be appropriate.

## Background

Effective cytoreduction at the time of primary surgery has been identified as the most important prognostic factor in the management of advanced-stage ovarian cancer [1, 2]. The goal of cytoreductive surgery is to obtain a macroscopic complete resection of the disease; such surgery is now considered to be the “real” optimal debulking surgery [3, 4]. Primary debulking surgery (PDS) followed by chemotherapy is the mainstay of treatment for advanced-stage ovarian cancer. However, for those unable to tolerate PDS or with an initial disease that is too extensive for optimal debulking, interval debulking surgery (IDS) following neoadjuvant chemotherapy (NAC) is considered to be a valuable alternative option for PDS. The usefulness of this strategy has been confirmed in a recent randomized trial performed by the European Organisation for Research and Treatment of Cancer Gynaecological Cancer Group and National Cancer Institute of Canada Clinical Trials Group; comparing PDS with IDS reported that progression-free survival and overall survival were similar in both groups [5].

Because of the anatomic proximity of the rectosigmoid to the female pelvic organs and its frequent involvement in ovarian cancer, rectosigmoid resection is the most frequently performed bowel surgery to achieve optimal cytoreduction [6]. Many investigators have studied the value of modified posterior exenteration, also known as low anterior en bloc resection or radical oophorectomy, during debulking surgery for ovarian cancer [6–9]. Although modified posterior exenteration has acceptable morbidity and mortality rates, anastomotic leakage after rectosigmoid resection remains a life-threatening potential complication [10].

If the tumor is suspected of having infiltrated the pelvic side wall, an en bloc resection of the pelvic side wall(s) together with the uterus, adnexa, and rectosigmoid should be performed to achieve optimal cytoreduction and to complete the surgical procedure safely [6, 11]. This surgical procedure is accompanied by the sacrifice of the ipsilateral autonomic nerves, resulting in postoperative bladder dysfunction to some extent [12]. In addition, as a pelvic side wall resection involves the removal of connective tissue containing lymphatic tissue around the internal iliac vessels and/or the internal iliac vessels themselves, possibly resulting in severe bleeding.

In ovarian cancer patients with tumor involvement of the cul-de-sac in whom a modified posterior exenteration with or without pelvic side wall resection has been performed, the histopathologic evaluation of tumor spreading is useful for evaluating the validity of the selection of this type of surgery. In the present study, we assessed the extents of tumor spreading in the rectosigmoid wall and pelvic side wall in modified posterior exenteration specimens from patients with primary ovarian, tubal, and peritoneal cancer during PDS and IDS. Then, we compared the validity of selecting this surgical procedure in the patients undergoing PDS with that in the patients undergoing IDS.

## Methods

**Fig. 1:**
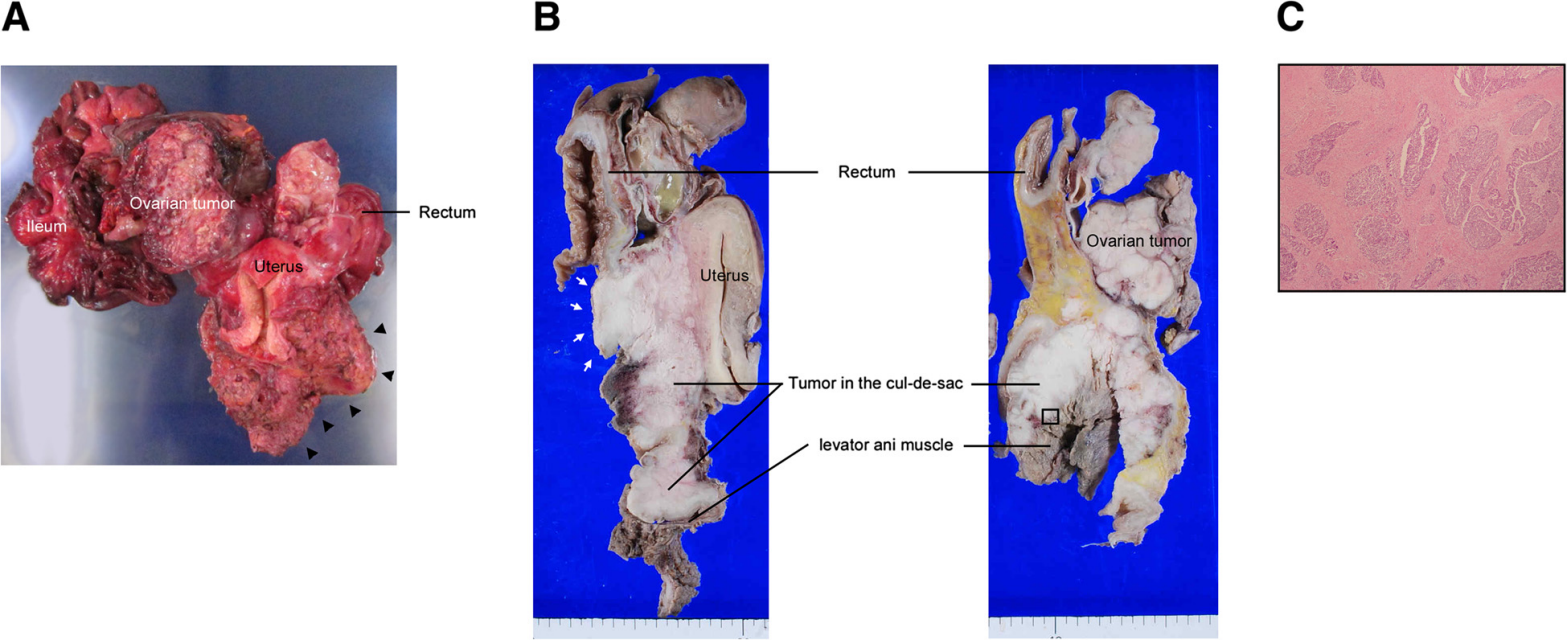
Surgical specimen of a posterior exenteration with the left pelvic side wall resection. **a** Ventral view of an en bloc resection specimen of the pelvic side wall tissue together with the uterus, adnexa, rectosigmoid, and ileum. The specimen included intact parietal pelvis tissue around the tumor spreading in the deep retroperitoneal space (*arrowheads*). **b** Cut surface of the specimen. Tumor infiltration into the mucosa of the rectal wall can be seen (*white arrows*). The framed rectangle corresponds to the area shown in Fig. 1c. **c** Histopathologic section of the specimen showed tumor infiltration into the skeletal muscle (hematoxylin and eosin staining, ×20)

All consecutive patients who underwent modified posterior exenteration as part of debulking surgery for primary ovarian, tubal, and peritoneal cancer at the Chiba University Hospital between April 2008 and March 2013 were evaluated for inclusion in this study. All the surgical procedures were performed by gynecologic oncologists working at our institution. We performed a laparotomy as an up-front surgery in all the cases with advanced-stage ovarian cancer. When PDS was thought to be possible, we then performed cytoreductive surgery. In patients with metastatic disease that was initially too extensive for optimal debulking, the abdominal wall was closed and NAC followed by IDS was considered. During modified posterior exenteration, preservation of the pelvic autonomic nerves on both sides was attempted whenever possible. However, an en bloc resection of the pelvic side wall(s) together with the uterus, adnexa, and rectosigmoid, resulting in the unilateral or bilateral sacrifice of the pelvic autonomic nerves, was necessary in cases with suspected tumor spreading in the pelvic side wall(s). The type of surgery was chosen by gynecologic oncologists depending upon the intraoperative findings of the disease in each patient. The tumor spreading was verified by inspection and manual palpation during the operation as well as by preoperative findings using various radiologic modalities including magnetic resonance imaging (MRI). Decisions regarding the proximal and distal resection sites of the rectosigmoid were made based on these findings. During IDS following NAC, if the masses were palpable under the pelvic peritoneum or the visceral serous membrane appeared to be intact, we made efforts to remove all of these tissues. Our surgical procedure for a modified posterior exenteration has been previously described [12]. Regarding the extent of the pelvic side wall resections, our technique corresponds to that of laterally extended endopelvic resection advocated by Höckel [11]. If the primary and metastatic tumors in the cul-de-sac had invaded laterally and posteriorly into the uterosacral ligament and the inferior hypogastric plexus was to be sacrificed, the cardinal ligament and the posterior leaf of the vesicouterine ligament were divided. In cases with tumor spreading in the deep retroperitoneal space, the lateral resection line is formed by following the lumbosacral nerve plexus, piriformis muscle, internal obturator muscle, and levator ani muscle. During surgery for these patients, an ipsilateral pelvic lymphadenectomy and a division of the internal iliac vessels were performed when the pelvic side wall resection was accomplished. Consequently, in surgical specimens of a modified posterior exenteration with pelvic side wall resection, tumor spreading in the side wall(s) was surrounded with the parietal pelvis tissue (Fig. 1).

The following data were retrieved from the patients’ medical records: patient age, primary site of disease, clinical stage, largest tumor size at the time of surgery, timing of surgery (PDS or IDS), type of surgery (modified posterior exenteration without pelvic side wall resection, with unilateral pelvic side wall resection, or bilateral pelvic side wall resection), final histopathologic results, and follow-up data. With respect to the histopathology, all the available information, including the histopathologic subtype of the tumor, the extent of tumor spreading in the rectosigmoid and pelvic side walls, and the margin status in the specimen, was recorded. Statistical analyses were performed using the Wilcoxon signed-rank test and the Fisher exact test. A value of *P* less than 0.05 was considered statistically significant.

This study protocol was approved by the Institutional Review Board of the Chiba University School of Medicine.

## Results

During the 5-year study period, modified posterior exenteration was performed in 75 patients with primary ovarian, tubal, and peritoneal cancer at the time of initial therapy. The clinical and tumor characteristics of all the patients in this series are shown in Table 1. PDS was performed in 38 patients (51 %), and IDS was performed in 37 patients (49 %). Colorectal anastomosis using a circular stapling device or a hand-sewn technique was performed in 69 patients (92 %). Hartmann’s operation was performed in five patients, and posterior pelvic exenteration accompanied by the resection of the vaginal posterior wall was performed in one. Modified posterior exenteration without pelvic side wall resection was performed in 58 patients (77 %). Modified posterior exenteration with unilateral and bilateral pelvic side wall resection was performed in 16 patients (21 %) and 1 patient, respectively. Among the patients in whom a modified posterior exenteration without pelvic side wall resection was performed, a pelvic lymphadenectomy was performed in 47 patients. As mentioned above, a pelvic lymphadenectomy was performed naturally in all 17 patients in whom a modified posterior exenteration with pelvic side wall resection was performed. Sixty-nine patients (92 %) had no visible tumor after surgery, and six other patients had residual tumors with a maximal diameter of <0.5 cm. None of the patients had macroscopic residual tumor in the pelvis.

**Table 1:** Patient and tumor characteristics

Characteristics	Result
Age, median (range), y	60 (31–83)
Diagnosis and stage	
Ovarian cancer (*n* = 53)	
IC	5
IIC	6
IIIB	1
IIIC	26
IV	15
Tubal cancer (*n* = 17)	
IIC	1
IIIB	2
IIIC	12
IV	2
Peritoneal cancer (*n* = 5)	
IIIC	4
IV	1
Histology, n (%)	
Serous	48 (64)
Clear cell	8 (11)
Endometrioid	7 (9)
Others	12 (16)
Largest size of pelvic tumor at surgery, *n* (%)	
<50 mm	22 (29)
50–100 mm	18 (24)
>100 mm	35 (47)
Pelvic lymph node involvement, *n* (%)	
Positive	26 (35)
Negative	38 (51)
No pelvic lymph nodes found	11 (15)

Table 2 shows the differences in the extent of tumor spreading in the rectosigmoid wall between the patients who underwent a modified posterior exenteration in the PDS group and those in the IDS group. Of the 38 patients who underwent PDS, the resection margins in the specimen were positive in two patients (5 %); both patients had positive distal (anal side) margins in the rectosigmoid because of tumor invasion in the lymphovascular space. Of the 37 patients who had undergone IDS, two patients (5 %) had positive resection margins; the distal margin in the rectosigmoid in one patient, and the proximal (oral side) margin in the rectosigmoid in the other patient. Two other patients (5 %) had close resection margins, which were defined as surgical margins within 5 mm of the tumor spread; the distal margin in the rectosigmoid in one patient, and the proximal margin in the rectosigmoid in the other patient.

**Table 2:** Differences in the extent of tumor spreading in the rectosigmoid wall between patients who underwent a modified posterior exenteration in the PDS group and those in the IDS group

Characteristics	PDS (*n* = 38)	IDS (*n* = 37)	*P* value
Rectosigmoid wall involvement			0.939
Positive	25	24	
Depth of tumor invasion			0.012
Mucosal or submucosal layer	7	3	
Muscular layer	11	5	
Serosal layer	7	16	
Negative	13	13	
Factor in rectosigmoid adhesion			0.706
Tumor involvement of the mesocolon	2	1	
Tumor involvement of the peritoneum of the cul-de-sac	4	2	
Fibrosis, necrosis, and/or granulation	3	8	
Endometriosis	3	1	
No histopathologic finding	1	1	

*PDS* primary debulking surgery, *IDS* interval debulking surgery after neoadjuvant chemotherapy

Table 3 shows the differences in the extent of tumor spreading in the pelvic side wall between the patients who underwent a modified posterior exenteration with pelvic side wall resection in the PDS group and those in the IDS group. None of the patients included in this study had positive or close circumferential or vaginal margins.

**Table 3:** Differences in the extent of tumor spreading in the pelvic side wall between patients who underwent a modified posterior exenteration with pelvic side wall resection in the PDS group and those in the IDS group

Characteristics	PDS (*n* = 12)	IDS (*n* = 5)	*P* value
Pelvic side wall involvement			0.354
Positive	8	2	
Extent of tumor spread			0.435
Parametrium and/or paracolpium	8	2	
Cardinal ligament and/or deep retroperitoneal space	3	1	
Ureter	1	0	
Internal iliac vessels	0	0	
Levator ani muscle	1	0	
Negative	4	3	
Pelvic lymph node involvement			1.000
Positive	5	2	
Negative	7	3	

The median duration of the follow-up period was 30.1 months (range, 6.2–74.5 months). Overall, 25 recurrences (33 %), 4 pelvic recurrences (5 %), and 12 deaths (16 %) occurred during the follow-up period. One of two patients who underwent PDS with positive resection margins in the rectosigmoid had multiple bone metastases as a recurrence. Among four patients who underwent IDS with positive or close resection margins in the rectosigmoid, one patient had a pelvic recurrence and additional two patients had distant metastases as a recurrence during this period.

## Discussion

The present study evaluated the histopathologic results of tumor spreading in surgical specimens obtained during modified posterior exenteration for primary ovarian cancer. Our results indicate that during surgery for ovarian cancer presenting with tumor involvement of the cul-de-sac, close inspection and palpation by gynecologic oncologists can enable the extent of tumor spreading in the pelvis to be estimated in most cases, enabling valid decisions as to whether an en bloc resection of the pelvic tumors together with the rectosigmoid and the pelvic side wall might or might not be appropriate.

Tumor involvement of the rectosigmoid wall was histopathologically confirmed in 65 % of the cases. A previous study reported that in 73 % of patients with suspected infiltration of the cul-de-sac (bulky tumor, disseminated tumor spread), the rectosigmoid wall was histopathologically infiltrated by the tumor [13]. They argued that in the majority of ovarian cancer patients with intraoperatively suspected cul-de-sac infiltration, residual tumor is likely to be present in the wall of the rectosigmoid if only deperitonealization, and no en bloc resection of the uterus and rectosigmoid, is performed. In this study, histopathologic examinations suggested that in cases without tumor spreading in the rectosigmoid or cul-de-sac, a modified posterior exenteration was performed because of fibrosis, necrosis, granulation, and/or endometriosis. Macroscopic inspection may overestimate the tumor involvement in such cases. MRI is an anatomic, high-resolution imaging modality that is widely used to guide the management of patients with ovarian cancer [14, 15]. Of course, our surgical decisions were made based not only on macroscopic inspection, but also on dynamic contrast-enhanced and diffusion-weighted MRI findings. The extent of tumor spreading in the rectosigmoid wall was deeper in the patients who underwent PDS than in those who underwent IDS. This difference was likely due to the effect of neoadjuvant chemotherapy before surgery, which reduced the tumor volume and spreading. However, a histopathologic evaluation showed that the frequency of tumor involvement of the rectosigmoid in patients who have undergone modified posterior exenteration during PDS was equivalent to that in the patients who have undergone modified posterior exenteration during IDS.

In this study, histopathologic examinations showed that the resection margins in the rectosigmoid wall were positive in 5 % of the patients who had undergone PDS, and were positive in 5 % and close (surgical margins ≤5 mm) in an additional 5 % of the patients who had undergone IDS. The lower segment of the rectum lies retroperitoneally. In the greater part of ovarian cancer cases presenting with a confluent tumor in the cul-de-sac, the lower segment of the rectum is free of tumor, since ovarian cancer grows along the peritoneal lining. Despite an intent to remove all visible and palpable tumors, however, there are some cases in which the resection margins are microscopically positive. In PDS, tumor-free resection margins (R0) are associated with a significant reduction in pelvic recurrences among ovarian cancer patients undergoing modified posterior exenteration [13]. It is not evident that all attempts should be made to achieve an R0 status during IDS. In the current series, the rate of positive resection margins in the rectosigmoid wall among the patients undergoing PDS was equivalent to that in patients undergoing IDS. Half of the patients with positive or close resection margins in the rectosigmoid experienced distant recurrence. This result may have been due to the wide-spread of tumor reflecting in positive or close resection margin in the rectosigmoid and microscopic residual disease remaining in extra-pelvic areas.

Gynecologic oncologists occasionally encounter cases with tumor spreading in the deep retroperitoneal space. Not only to secure negative surgical margins but to accomplish the procedures safely and securely for cases, the pelvic side wall resection is needed for these cases. In the current series, tumor involvement of the parametrium and/or the parametrium surrounding the uterus and vagina was frequent (75 % of the patients in the PDS group and 40 % of the patients in the IDS group). Lateral tumor spreading to the cardinal ligament and/or deep retroperitoneal space was histopathologically confirmed in 25 % of the patients in the PDS group and in 20 % of the patients in the IDS group. In addition, tumor spreading beyond the deep retroperitoneal space to the levator ani muscle was confirmed in only one patient in whom a pelvic side wall resection had been performed. Similarly, the previous study showed that though infiltration of the parametrium and paracolpium frequently occurred in patients who underwent a modified posterior exenteration with pelvic side wall resection, tumor involvement of the parietal pelvis, including the internal iliac vessels, levator ani muscle, and internal obturator muscle, was rare [11, 16]. Although nerve-sparing modified posterior exenteration on at least one side is useful, pelvic side wall resection results in the sacrifice of the pelvic autonomic nerves to some extent [12]. The results of this study showing that none of the patients with pelvic side wall resection had positive or close circumferential margins in the specimens suggest the validity of this surgery.

From the results of this study, the presence of cancer cells within desmoplastic stroma was histopathologically demonstrated in the palpable masses under the pelvic peritoneum, and the visceral serous membrane appeared to be intact during IDS in many cases. The use of NAC before surgery induces tumor necrosis, fibrosis, macrophage infiltration, and tumor-induced inflammation in the peritoneal cavity. Then, residual cancer cells remain in deep visceral and retroperitoneal tissue. There is a report that among advanced ovarian cancer patients with residual disease of <1 cm, the size of the viable tumor in the operative specimens was inversely correlated with progression-free survival and overall survival [17]. We supposed that the removal of as many palpable masses under the peritoneum as necessary was feasible. A recent report has suggested that microscopically carcinomatous areas often have a benign visual appearance after neoadjuvant chemotherapy in advanced ovarian cancer [18]. Accordingly, intraoperative macroscopic evaluation of the extent of the tumor is more accurate in PDS than in IDS. It was stated that the removal of all peritoneal surfaces affected at the time of diagnosis should be considered in IDS. However, this approach would lead to more extensive debulking operations, with the consequent loss of some of the benefits that are presently linked with IDS. Thus, a study investigating whether the resection of sites involved by primary and metastatic tumors at the time of diagnosis are associated with a higher survival should be performed in larger prospective trials in the future.

There are some limitations that must be considered when interpreting these data. First, this was a retrospective study design. Second, the number of the patients whom pelvic side wall resection had been performed was small and further studies with the long follow-up period are needed to confirm the efficacy of this surgical procedure. Despite these limitations, study strengths consist of a report from one institution where the treatment approaches were not changed throughout the inclusion period.

## Conclusions

The evaluation of whether the tumor involves the rectosigmoid wall and the pelvic side wall is critical, based on physical investigations performed during the modified posterior exenteration with or without pelvic side wall resection. As a close inspection and palpation by gynecologic oncologists to estimate the extent of tumor spreading in the pelvis seems to be appropriate, this procedure might be achieved under a conventional laparotomy. This approach may also be useful for the cytoreduction of the upper-abdominal spreading of disease in cases of advanced-stage ovarian cancer.

## Consent

Written informed consent was obtained from the patient for the publication of this report and any accompanying images.
